# APDS in a 3-year-old boy presenting with EBV viremia and hodgkin lymphoma associated with a novel germline heterozygous variant in PIK3CD and with characteristic immune phenotype but no upregulation of the T cell mTOR pathway

**DOI:** 10.1186/s13223-026-01014-4

**Published:** 2026-03-17

**Authors:** Devyani Bakshi, Andrew Wong-Pack, Stacey Marjerrison, Rae Brager, Manish J. Butte, Timothy J. Thauland, Jenny Garkaby

**Affiliations:** 1 Division of Clinical Immunology, Allergy and Dermatology, Department of Pediatrics, McMaster University, McMaster Children’s Hospital, Hamilton, ON Canada; 2 Division of Pediatric Hematology/Oncology, Department of Pediatrics, McMaster University, McMaster Children’s Hospital, Hamilton, ON Canada; 3 Department of Pediatrics, Division of Immunology and Allergy, University of California Los Angeles, Los Angeles, CA USA

**Keywords:** PIK3CD, APDS, EBV viremia, Hodgkin lymphoma

## Abstract

**Background:**

Activated phosphoinositide 3-kinase δ syndrome (APDS) is an inborn error of immunity in the *PIK3CD* gene caused by an increase in phosphoinositide 3-kinase δ (PI3Kδ) activity. APDS is characterized by immune dysregulation such as senescent T cells, lymphoproliferation, and hypogammaglobulinemia. Patients present with lymphoid hyperplasia, recurrent sinopulmonary infections, recurrent viremias, and lymphomas.

**Case presentation:**

We present a case report of a patient with APDS due to a novel variant in the *PIK3CD* gene. Our patient was identified at 3 years of age due to persistent EBV viremia and Hodgkin lymphoma. Immune evaluation demonstrated upregulation of T follicular helper cells and CD10 + B cells were consistent with the lymphocytic phenotype present in patients with APDS. However, there was no upregulation of the T cell mTOR pathway by functional testing. The patient was found to carry a novel variant, (c.58G > A p.(Val20lle)), in the *PIK3CD* gene.

**Conclusions:**

We describe a 3-year-old patient with a novel variant in the *PIK3CD* gene (c.58G > A p.(Val20lle)) presenting with EBV viremia, Hodgkin lymphoma, upregulation T follicular helper cells and CD10 + B cells consistent with a phenotype of APDS in a 3-year-old boy. This case broadens our understanding of the genetic and phenotypic spectrum of *PIK3CD* gene mutations in APDS.

**Supplementary Information:**

The online version contains supplementary material available at 10.1186/s13223-026-01014-4.

## Background

Activated phosphoinositide 3-kinase δ syndrome (APDS) is an inborn error of immunity caused by an increase in phosphoinositide 3-kinase δ (PI3Kδ) activity [1]. APDS1 is phenotypically classified under the “predominantly antibody deficiencies” category as per the International Union of Immunological Societies (IUIS) expert committee (EC) on inborn errors of immunity [2]. APDS1 is caused by a gain of function (GOF) mutation in the *PIK3CD* gene, which encodes for the p110δ catalytic subunit of PI3Kδ [1, 3]. The *PIK3CD* gene is expressed predominately in hematopoietic stem cells, lymphocytes, and myeloid cells [4]. PI3Kδ is part of a family of phosphoinositide 3-kinases (PI3K) that play a role in cell growth, proliferation, and survival [3]. Each PI3K is comprised of catalytic and regulatory subunits, which act on downstream pathways responsible for the regulation of cell proliferation, differentiation, and apoptosis [1]. APDS1 is characterized by senescent T cells, lymphoproliferation (both malignant and non-malignant), hypogammaglobulinemia, and/or elevation of IgM [1, 3, 5].

APDS patients exhibit features of immune dysregulation, such as immune thrombocytopenia (ITP), autoimmune hemolytic anemia, splenomegaly, lymphoid hyperplasia, and glomerulonephritis [5–7]. Patients often have severe sinopulmonary infections, leading to bronchiectasis in over half of APDS patients [5, 6]. Other features include recurrent viremias (i.e. Epstein-Barr virus (EBV), cytomegalovirus (CMV), herpes simplex virus (HSV) and malignancy, especially lymphomas [1, 4, 6, 7].

The most common malignancy associated with APDS is B cell lymphoma [5]. This includes Hodgkin lymphoma, diffuse large B-cell lymphoma, marginal zone B-cell lymphoma, and mucosal-associated lymphoid tissue-derived lymphoma [5]. The B-cell lymphoma rate in APDS patients is variable and has been reported as approximately 13–30% [5–7]. The median age at lymphoma presentation is also variable in patients with APDS, ranging between 6 and 59 years of age [5].

APDS1 is a rare and probably under-reported and under-recognized condition. We report a novel variant of unknown significance resulting in increased PI3Kδ activity and a phenotype of APDS.

## Methods

### Immunophenotyping

**Fig. 1:**
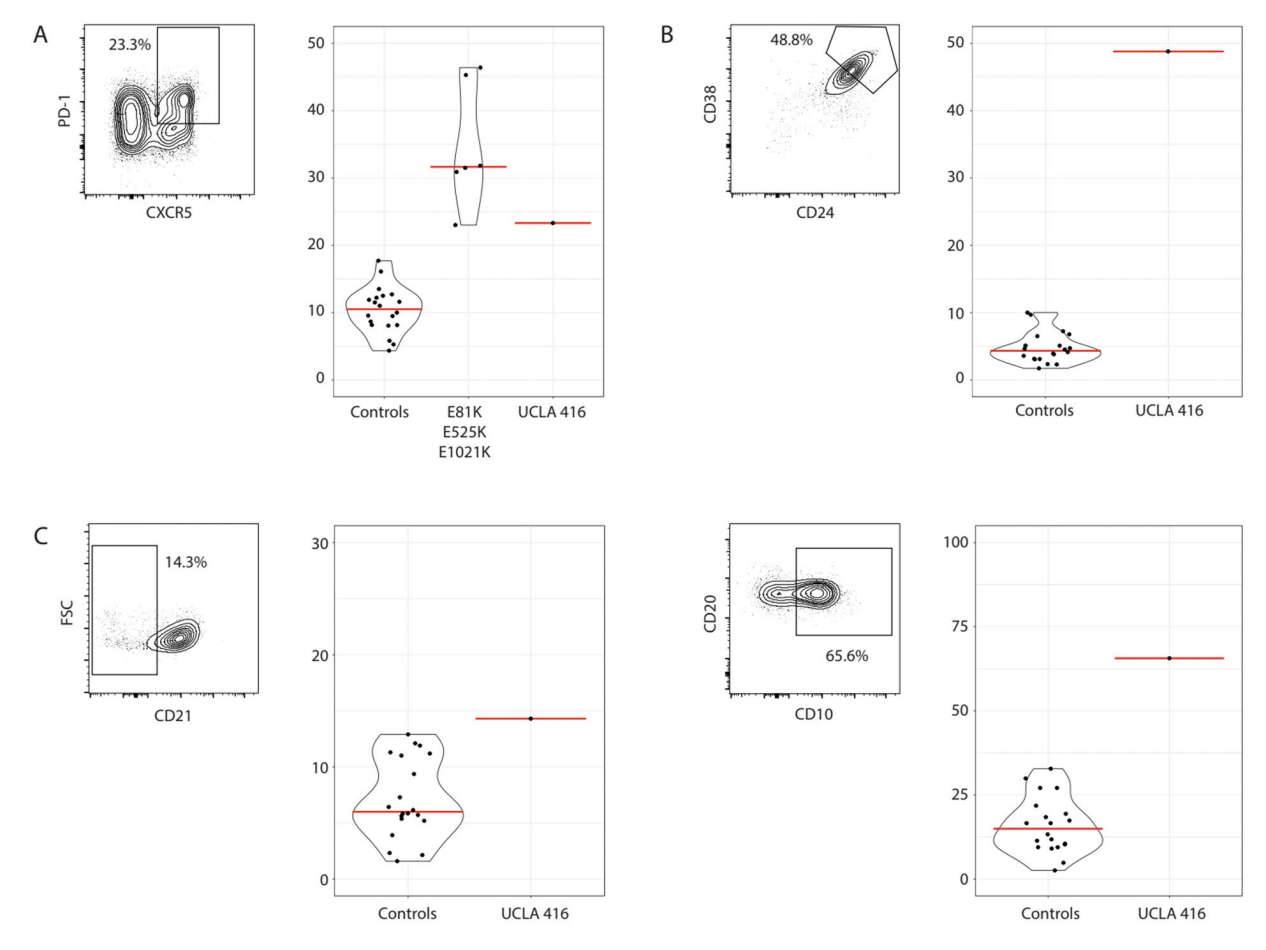
**A** Percentage of T follicular helper (Tfh) cells among CD3 + CD4+CD45RO+ cells were determined by flow cytometry of PBMCs. The result is compared to healthy controls and known APDS patients. **B**, **C** Percentage of CD24 + + CD38 + + transitional **B**, CD21 low immature **C**, or CD10 + cells among CD19 + CD20+CD27-B cells. Results are compared to healthy controls

Flow cytometry was conducted with three panels of antibodies to identify T cell, Helper T cell, and B cell subsets among peripheral blood mononuclear cells (PBMCs). PBMCs were incubated with Fc block and then labeled with antibody cocktails on ice in FACS buffer (PBS supplemented with 2% FBS and 1 mM EDTA), washed, and analyzed by FACS. Data analysis was conducted in FlowJo to identify the following T cell and B cell populations. *T cells*: Naive (CD45RA+CCR7+), Central Memory (CD45RA-CCR7+), Effector Memory (CD45RA-CCR7-), TEMRA (CD45RA+CCR7-), Activated (HLA-DR+), Exhausted (PD-1+) and Senescent (CD57+) subsets were identified for both CD4 (CD3 + CD4+) and CD8 (CD3 + CD8+) T cells. *Helper T cells*: Tfh (CCR5 + PD-1+), Th1 (CCR6-CXCR3 + CCR4-), Th2 (CCR6-CXCR3-CCR4+), Th17 (CCR6 + CXCR3-CCR4+), and Th1_17 (CCR6 + CXCR3+CCR4-) subsets were identified among CD4 memory T cells (CD3 + CD4+CD45RO+). *B cells*: Naive (CD27-), Immature (CD27-CD21 low), Transitional (CD27-CD24 + + CD38++), Memory (CD27+), Unswitched Memory (CD27 + IgM + IgD+), and Switched Memory (CD27 + IgM-IgD-) subsets were identified among CD19 + CD20+ B cells (Fig. 1).

## Phosflow assay

PBMCs were incubated on ice for 10 min with 5 µg/mL anti-CD3ε in complete T cell medium, washed, and incubated for 20 min at 37 deg C with 10 µg/mL F(ab’)_2_ goat anti-human IgM and 20 µg/mL goat anti-mouse IgG to crosslink B cell and T cell receptors respectively. Cells were fixed for 10 min at 37 deg C with an equal volume of pre-warmed Cytofix Buffer (BD Biosciences), washed with FACS buffer, and incubated with Fc block, anti-CD19, and anti-CD4. After surface staining, cells were permeabilized with Perm Buffer III (BD Biosciences) pre-chilled to -20 deg C for 30 min on ice, washed with FACS buffer, and incubated with anti-pAkt and anti-pS6 for 30 min at RT.

## Antibodies

For immunophenotyping, the following antibodies were purchased from Biolegend: Human TruStain FcX; *B cell panel*: CD19 APC-Cy7 (HIB19), CD20 BV711 (2H7), CD24 AF488 (ML5), CD10 PE (HI10a), CD21 PerCP-Cy5.5 (Bu32), CD38 PE-Cy7 (HB-7), IgM BV605 (MHM-88), IgD APC (IA6-2); *T cell and Helper T cell panels*: CD3 BV605 (OKT3), CD4 PerCP-Cy5.5 (RPA-T4), CD8 PE (RPA-T8), HLA-DR AF488 (L243), CCR7 PE-Cy7 (G043H7), CD45RA BV421 (HI100), PD-1 AF647 (EH12.2H7), CD57 BV785 (QA17A04), CD45RO APC-Cy7 (UCHL1), CXCR5 PE (J252D4), CCR6 AF488 (G034E3), CXCR3 PE-Cy7 (G025H7), CCR4 BV421 (L291H4). For phosflow, functional grade anti-CD3ε (clone OKT3), CD19 BV421 (HIB19), and CD4 BV605 (OKT4) were from Biolegend; pAkt Ser473 AF647 (D9E), and pS6 Ser240/244 AF488 (D68F8) were from Cell Signaling Technology; F(ab’)_2_ goat anti-human IgM (109-006-129) and goat anti-mouse IgG (115-005-062) were from Jackson Immunoresearch.

## Case presentation

The proband is a Caucasian 3-year-old boy born to non-consanguineous parents following an uncomplicated pregnancy and delivery. Family history was non-contributory for immunodeficiency, autoimmunity, or malignancy.

He was first referred to the hematology/oncology team at our center for a 1.5-year history of fluctuating, unilateral left-sided cervical lymphadenopathy. The immunology and infectious disease services also became involved along the course of his workup.

On initial physical exam the patient had isolated bilateral cervical lymphadenopathy, which was non-erythematous, firm, rubbery and fixed in nature. His parents did not report any short stature, neurodevelopmental or growth delays that are sometimes also present in patients with APDS. He had been trialled on a course of oral antimicrobials with no improvement in his lymphadenopathy prior to his referral.

## Diagnostic assessment

Complete blood count (CBC) revealed microcytic anemia with a hemoglobin of 95 g/L, a mean corpuscular volume (MCV) of 67 fL, and slightly elevated reticulocyte count of 28%. Iron studies were consistent with iron deficiency anemia as outlined in Table 1.

Infectious workup was negative for Bartonella, Toxoplasma, and Blastomycosis. Further investigations revealed EBV viral capsid antigen (VCA) IgG was positive, but EBNA IgG was negative, indicative of recent infection. Quantitative EBV viral PCR revealed EBV viremia of 26,263 IU/mL, which gradually increased to 77,550 IU/mL within one month of follow-up (Table 1). Repeat EBV serology was again positive for VCA IgG and negative EBNA IgG, indicating lack of EBV clearance. C-reactive protein (CRP) was elevated at 60.9 mg/dL (normal: <10 mg/dL).

Lymphocyte immunophenotyping showed normal CD3+, CD3 + CD4+, CD3 + CD8+ cells. Natural killer cells were mildly elevated. B cell immunophenotyping revealed low naive B cells, low non-switched memory B cells, but normal IgM memory B cells, and normal switched memory B cells.

Immunoglobulins were grossly normal except for a mild elevation in IgA. Rubella, Mumps Diphtheria and Tetanus titres were protective. Measles and varicella zoster virus serology were non-reactive (Table 1).

**Fig. 2:**
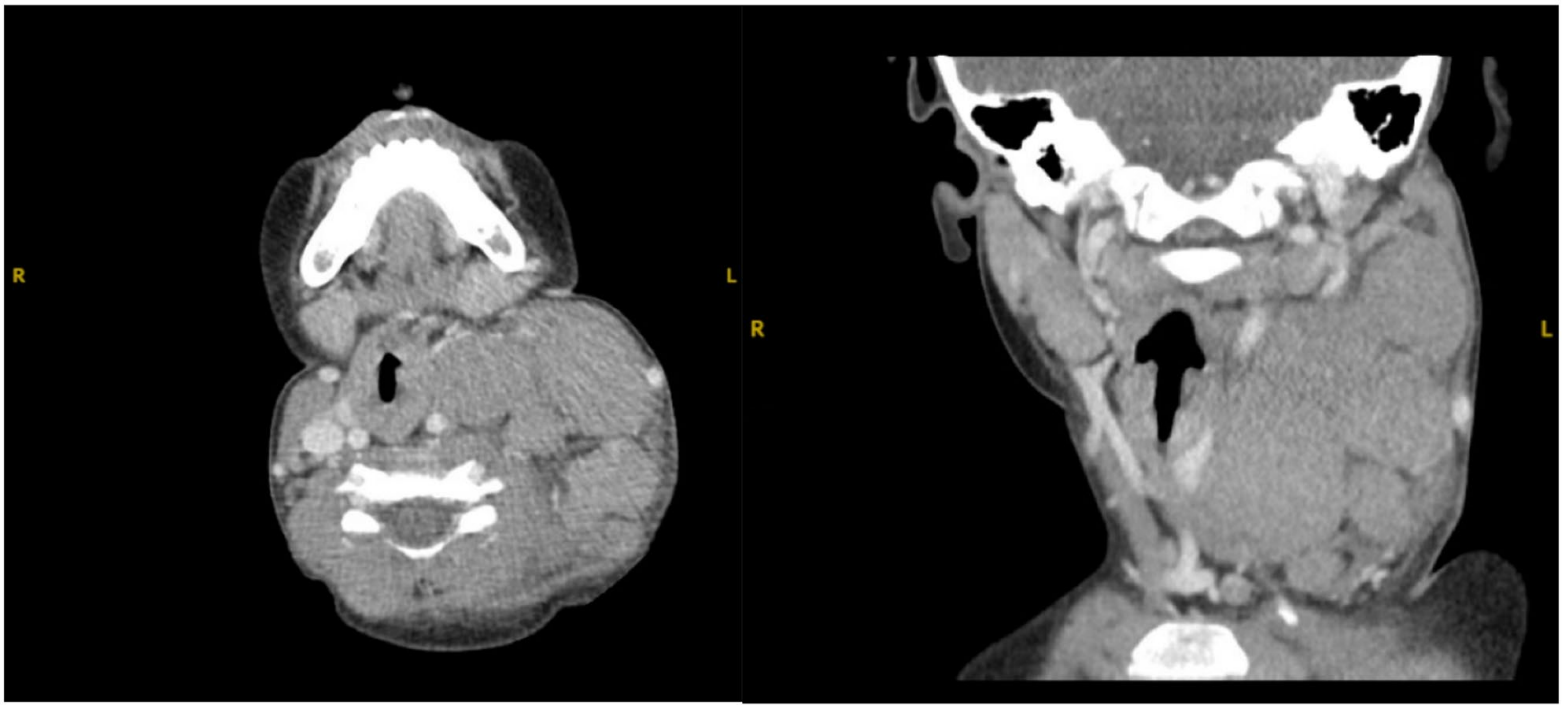
Computed Tomography Neck with Contrast: Axial and Coronal Views showing a large conglomerate of lymph nodes (11.5 cm) involving the left neck with significant mass effect

Repeated neck ultrasound studies showed growth of the cervical lymph nodes from 8 mm x 8 mm x 4 mm in size to the largest, measuring 22 × 30 × 30 mm within 8 months, confirmed by neck CT (Fig. 2). Clinically, his lymphadenopathy was still fluctuating and persistent. Despite the persistent EBV, it was decided to proceed with a lymph node biopsy to rule out malignancy. The lymph node biopsy of his cervical node revealed classical Hodgkin lymphoma, mixed cellularity type; a PET scan and bone marrow biopsy confirmed stage II disease.

**Fig. 3:**
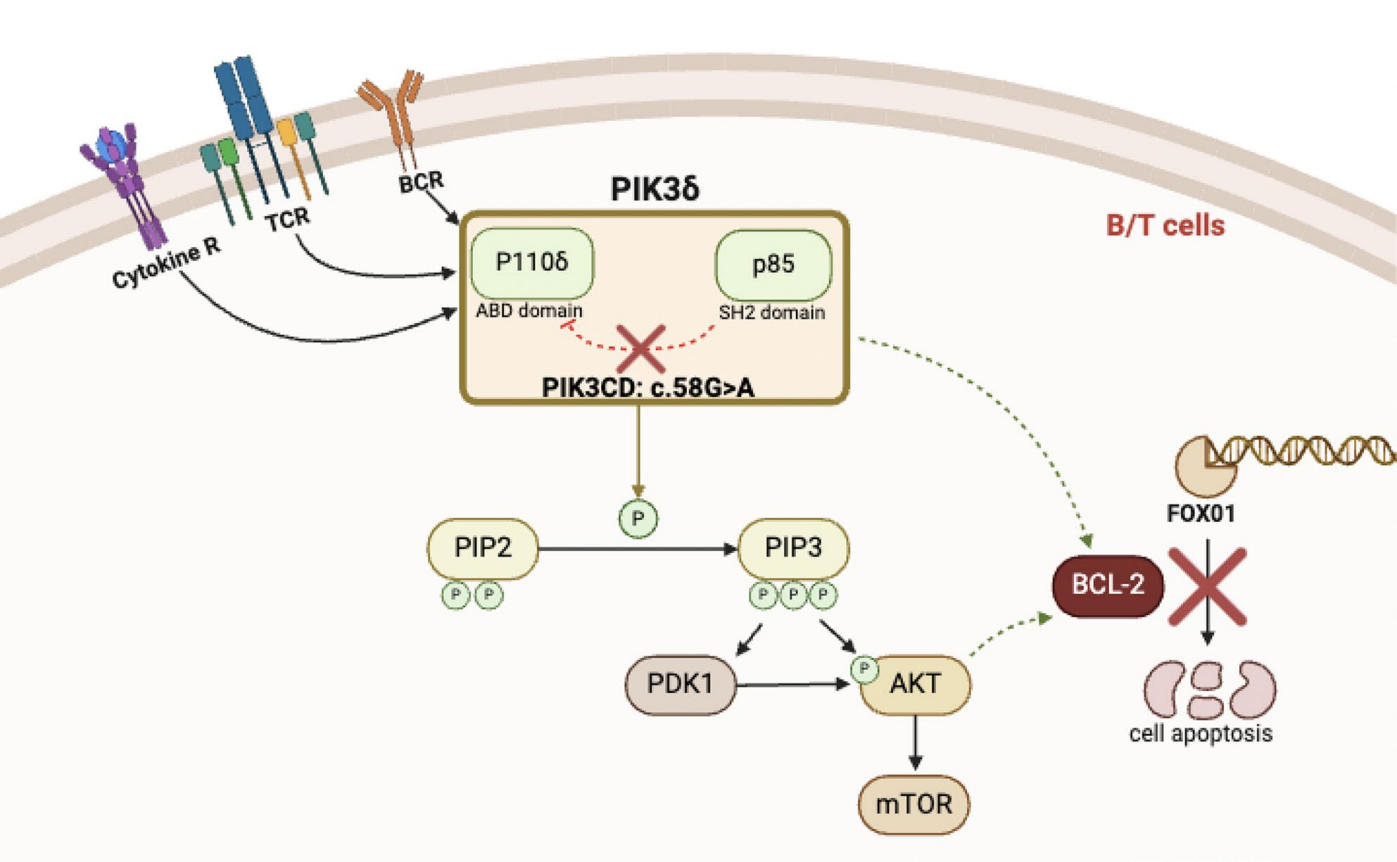
Schematic representation of PIK3δ activation pathway and downstream effects. The position of our proband’s variant has been marked with respect to the functional domains

A genetic panel targeted at inborn errors of immunity revealed a heterozygous variant of uncertain significance (VUS) in the *PIK3CD* gene (NM_005026.3:c.58G > A, p.Val20lle). This variant has not been previously described in the literature (Fig. 3). This variant in the *PIK3CD* gene is present in 0.0065% of the presumed healthy population according to large population databases (GenomAD). Familial segregation assay revealed both the proband’s father and 5-year-old sister bear the same variant. Neither the patient’s father nor sister had any features of autoimmunity, recurrent infections (including current EBV infection), or immune dysfunction on clinical history. A functional assay examining T cell and B cell subsets and the PI3K/AKT pathway was completed. Elevation of T follicular helper cells (T_FH_) of 25% and an increase in CD10 + B-cells (65%) were observed, which are consistent with findings seen in other patients with APDS. The mammalian target of rapamycin (mTOR) pathway was normal and not upregulated in T cells.

The patient underwent chemotherapy according to the GPOH-HD-2002 Protocol, intermediate risk, with two cycles of OEPA (vincristine, etoposide, prednisone, and doxorubicin) and two cycles of COPDAC (cyclophosphamide, vincristine, prednisone and dacarbazine) [8]. He had complete PET response after the first two cycles of therapy and did not require radiotherapy. He cleared EBV following the first cycle of chemotherapy.

Upon completion of chemotherapy and determination of genetic findings, our patient was referred for hematopoietic stem cell transplantation.

### Discussion and conclusions

We present a case of a three-year-old boy with persistent EBV viremia and Hodgkin lymphoma, associated with a variant of unknown significance in the *PIK3CD* gene and a functional immune profile consistent with findings seen in patients with APDS.

The *PIK3CD* gene encodes the catalytic subunit, p110δ catalytic subunit of PI3Kδ which is implicated in APDS1. PI3Kδ promotes the degradation of forkhead box O1 (FOXO1) transcription factors and ultimately leads to the upregulation of anti-apoptotic BCL-2 proteins and therefore, the increased survival of lymphoid cells [9]. PI3Kδ also promotes the activation of mTOR through the serine/threonine kinase AKT, and phosphoinositide-dependent kinase 1 (PDK1) [10]. Upregulation of the PI3Kδ/AKT/PDK1 signalling pathway leads to decreased cell apoptosis, resulting in the accumulation of exhausted B and T cells, lymphoproliferation, and decreased production of high-affinity antibody-producing B cells [6].

**Fig. 4:**
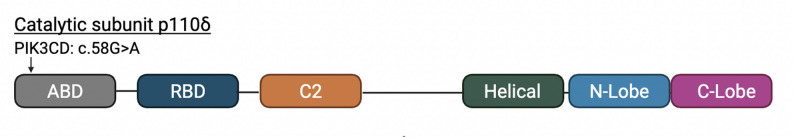
Schematic protein structure of catalytic P110δ and regulatory p85α subunits encoded by *PIK3CD*. Our proband’s variant is believed to impair the inhibitory contact of p85α resulting in hyperactivation of PI3Kδ [3]and an APDS phenotype. ABD: adaptor-binding domain (ABD); RBD: RAS GTPase binding domain (RBD); C2: C2 domain; Helical: Helical domain; N-lobe: amino side lobes; C- lobe: carboxyl side lobe. Image generated using Biorender.com

The p110δ catalytic subunit is composed of five primary functional domains: adaptor-binding domain (ABD), RAS GTPase binding domain (RBD), C2 domain, Helical domain, and lipid kinase domain [11]. The kinase domain is further separated into amino (N) and carboxyl (C) side lobes and is the site of E1021K, the most common pathogenic variant in APDS1 (Fig. 4) [5, 12, 13]. The ABD domain plays an important role in binding the regulatory p85 subunit, which is involved in inhibiting the C2, helical, and lipid kinase domains of p100δ [11]. A previously reported variant in the ABD domain was found to impair inhibitory contact of p85 resulting in hyperactivation of PI3Kδ [3] and an APDS phenotype (Fig. 3) [11].

While the true prevalence of APDS is unknown, it was suggested that certain APDS variants have high penetrance [6, 14]. In a large cohort study by *Coulter et al.*., of the 53 participants studied (from 30 APDS1 families), only one heterozygous individual with the E1021K variant had no reported health concerns [6, 14]. In our case, the father and five-year old sister were asymptomatic despite carrying the p.Val20lle variant. However, the patient’s sister did have borderline T_FH_ cells; elevation of T_FH_ can be a hallmark of APDS, which was also seen in the proband. A recent study by *Stewart et al.*., demonstrated that allele-specific expression due to epigenetic regulation can lead to variable expression of disease-causing alleles which can lead to phenotypic heterogeneity [15]. Therefore, it is possible that environmental/infectious factors, such as EBV, function as an immunologic trigger in a genetically predisposed host [15]. This explanation could support why in our case, the proband had symptoms of APDS while the father and older sister were asymptomatic despite carrying the p.Val20lle variant. This highlights the need for further research into the effect of epigenetic regulatory factors, both genetic and environmental, on APDS disease expression.

In our patient’s variant, a missense mutation leads to replacement of valine with isoleucine, both neutral and non-polar amino acids, at codon 20 of the *PIK3CD* gene. On his functional analysis of the *PIK3CD* variant, upregulation of T follicular helper cells and CD10 + B cells were consistent with the lymphocytic phenotype present in patients with APDS [10, 16]. Based on our data and the American College of Medical Genetics and Genomics (ACMG) VUS pathogenicity guidelines, the VUS in our patient p.Val20lle can now be classified as pathogenic as it satisfies PP4, PS3 criteria [17]. The ACMG VUS pathogenicity guidelines describe PP4 pathogenic criteria as highly specific patient phenotype for a disease with a single genetic etiology [17]. The PS3 criteria outline that there is in-vitro or in-vivo functional studies supportive of a damaging effect of the gene/gene product [17]. Function data on Franklin classified the variant as pathogenic, consistent with a missense variant in a gene that otherwise has low rate of benign missense mutation in which a missense mutation is likely associated with disease (Franklin). Although the in-silico prediction from Combined Annotation Dependent Depletion (CADD) score is 17.7, and from PolyPhen is 0.003, the clinical history and functional assay fulfil PP4, PS3 criteria of pathogenicity (Table 2).

Interestingly, the mTOR activation pathway was not upregulated on functional analysis. mTOR is both activated by PI3Kδ, and itself activates AKT [3]. Upregulation of mTOR would be expected in someone with GOF mutations in the *PIK3CD* gene. A possible explanation would be the chemotherapy and steroids received for the patient’s Hodgkin lymphoma which contributed to suppression of the mTOR pathway, although there was at least a two-month interval between the patient’s last chemotherapy cycle and the time of the functional assay.

Stone et al.., have identified a pathway linking PI3Kδ with TFH development in T cells, in which increased PI3Kδ caused inactivation of FOXO1 through AKT, causing enhanced T follicular cell (T_FH_) differentiation [18]. Thauland et al., described a patient with a novel APDS variant, Y524S, who had an excessive proportion of naive T_FH_ cells in the periphery and lymph nodes causing the accumulation of germinal centre B cells due to improper T_FH_ differentiation, not solely caused by enhanced activation of AKT [10]. Based on these findings, we suspect that the lymphoma in our proband may be due to germinal centre B cell accumulation secondary to inappropriate T_FH_ differentiation.

Hodgkin lymphoma has bimodal distribution and has an incidence rate of 2.6 per 100,000 cases in the United States [5, 13, 19]. The age of diagnosis is most often 20–40 years, followed by another peak at age 55 years and older [5]. Hodgkin lymphoma has been previously associated with APDS in older patients [5, 9, 10, 19]. Rates of lymphadenopathy and neoplasm are much higher in patients with APDS, approximately 13% for APDS1 and 28% for APDS2, when compared to the general population [5, 6, 9]. We present an unusual case of early onset Hodgkin lymphoma in a 3-year-old boy. The report expands that association, suggesting that Hodgkin lymphoma could be a primary disease manifestation of APDS in pediatric patients, even in the absence of other features of APDS such as recurrent infections or autoimmune manifestations.

There are several hypothesized mechanisms for the predisposition of B cell lymphomas in APDS patients. PI3Kδ is strongly expressed in B cells and has a role in B cell maturation; therefore, the uncontrolled activation of PI3Kδ/AKT/PDK1 pathway can lead to exhausted B cells that more predisposed to malignant transformation [5, 9].

APDS is generally characterized by generalized proliferation of lymphoid tissue and does not demonstrate predilection for one lymphoid region over the other [6, 7]. In the case we present, the patient presented with isolated cervical lymphadenopathy. This may be influenced by the focal nature of Hodgkin lymphoma and EBV viremia, which are both known to clinically present in cervical and upper body lymph node regions [8, 19]. Additionally, EBV infection can independently upregulate expansion of CD10 + B cells [20]. Therefore, the CD10 + B cell upregulation seen in the patient may be attributable to both APDS-associated immune dysregulation and/or germinal center proliferation secondary to EBV infection.

Overactivation of the PI3Kδ signalling pathway also leads to impaired cytotoxicity of NK and CD8 + T cells, which are vital for cancer surveillance [13]. The T cell phenotype in APDS is characterized by a decrease in CD4 + and CD8+ naïve T cells and an expansion in C8D+CCR7-CD45RA+ effector memory T cells [13]. Despite a normal or even high frequency of EBV-specific CD8 + T cells in APDS patients, the CD8 + T cells in APDS patients have an immunosenescent phenotype as demonstrated by impaired clonal expansion, defective killing of EBV-infected targets, and increased susceptibility to restimulation-induced cell death (RICD) [13].

In summary, this report describes a 3-year-old male with a novel missense variant in the *PIK3CD* gene that results in the amino acid change p.Val20lle, who developed EBV viremia and Hodgkin lymphoma. This case contributes to the spectrum of variants known in *PIK3CD*, both genotypically and phenotypically, in a rare, often under-recognized, inborn error of immunity. We suggest maintaining a high index of suspicion for EBV susceptible immunodeficiencies including APDS, even in the absence of other typical manifestations, in pediatric patients who present with malignancy. Furthermore, APDS should be considered in the workup of those patients regardless of family history, as penetrance could be incomplete.

*Ethics and consent to participate*: Consent to share information about the patient and his care was obtained from the patient’s parents.

**Table 1:** Investigations and lab values

Investigation	Reference range	Patient value
CBC		
Leukocytes	5.5–15.5 × 10^9^/L	15.2 × 10^9^/L
Hemoglobin	105–150 g/L	95 g/L
Hematocrit	0.320–0.460 L/L	0.308 L/L
Erythrocyte mean corpuscular volume (MCV)	73–89 fL	67 fL
Iron studies		
Iron	2.5–26.9 µmol/L	1.8 µmol/L
Iron saturation	0.2–0.5 SI%	0.03 SI%
Iron binding capacity	54-79µmol/L	56 µmol/L
Infectious serology		
Bartonella Henselae IgG	< 1:64	Non-reactive
Toxoplasma Gondii IgG		Non-reactive
Blastomycosis Dermatitidis A band		Non-reactive
Borrelia Burgdorferi IgG and IgM		Non-reactive
Epstein Barr Virus Capsid Antibody IgM		Non-reactive
Epstein Barr Virus Nuclear Antibody IgG		Non-reactive
Epstein Barr Virus Capsid Antibody IgG		Reactive
Epstein Barr virus quantitative PCR		
April 3, 2023		26, 263 IU/mL plasma
May 15, 2023		77, 550 IU/mL plasma
May 26, 2023		91, 300 IU/mL plasma
June 5, 2023		36, 850 IU/mL plasma
Lymphocyte immunophenotyping		
CD3 + Mature	1.4–3.7 × 10^9^ L	2.09 × 10^9^ L
CD3 + CD4+	0.7–2.2 × 10^9^ L	0.98 × 10^9^ L
CD3 + CD8+ T suppressor cells	0.49–1.3 × 10^9^ L	0.87 × 10^9^ L
CD4/CD8 ratio	1.0-2.1	1.1
CD19+	0.37–1.34 × 10^9^ L	0.55 × 10^9^ L
CD3-CD16 + CD56+	0.13–0.72 × 10^9^ L	0.86 × 10^9^ L
B cell subsets		
naive B cells	120–1000 × 10^6^/L	114 × 10^6^/L
non-switched memory B cells	8.4–220 × 10^6^/L	6 × 10^6^/L
IgM memory B cells	0.6–100 × 10^6^/L	4.4 × 10^6^/L
switched memory B cells	2.2–250 × 10^6^/L	2.6 × 10^6^/L
Transitional B cells	11–270 × 10^6^/L	76.9 × 10^6^/L
Plasmablasts	0.3–36 × 10^6^/L	11. 3 × 10^6^/L
Immunoglobulins		
IgG	4.53–9.16 g/L	8.72 g/L
IgA	0.20–1.00 g/L	1.18 g/L
IgM	0.19–1.49 g/L	0.79 g/L
Vaccine titres		
Rubella IgG	Reactive	Reactive
Mumps IgG	Reactive	reactive
Measles IgG	Reactive	Non-reactive
Anti-Diphtheria toxoid IgG	> = 0.1 IU/mL	0.25 IU/mL
Anti-Tetanus toxoid IgG	> = 0.1 IU/mL	0.71 IU/mL
Varicella zoster virus IgG	Reactive	Non-reactive

**Table 2:** In-silico prediction models for the p.Val20lle variant

In-silico prediction model tool	Output
CADD Score	17.7
PolyPhen	0.003
SIFT	1.00

PI3Kδ promotes the activation of mTOR through the serine/threonine kinase, AKT, and phosphoinositide-dependent kinase 1 (PDK1). PI3Kδ also promotes the degradation of forkhead box O1 (FOXO1) transcription factors and leads to the upregulation of anti-apoptotic BCL-2 proteins. Image generated using Biorender.com.

## Supplementary Information

Below is the link to the electronic supplementary material.

Supplementary Material 1

## Data Availability

Data sharing is not applicable to this article as no datasets were generated or analysed during the current study.

## References

[1] Singh A, Joshi V, Jindal AK, Mathew B, Rawat A. An updated review on activated PI3 kinase delta syndrome (APDS). Genes Dis. 2020;7(1):67–74. 32181277 10.1016/j.gendis.2019.09.015 PMC7063426

[2] Bousfiha AA, Jeddane L, Moundir A, Poli MC, Aksentijevich I, Cunningham-Rundles C et al. The 2024 update of IUIS phenotypic classification of human inborn errors of immunity. J Hum Immun. 2025;1(1):e20250002. 10.70962/jhi.20250002 PMC12829316 41608113

[3] Hanson EP, Uzel G, Hambleton S. Key pathways in primary immune deficiencies. Stiehm’s immune deficiencies. Elsevier; 2020. pp. 99–114.

[4] Shashaani N, Chavoshzadeh Z, Ghasemi L, Ghotbabadi SH, Shiari S, Sharafian S, et al. Immunodeficiency due to a novel variant in PIK3CD: a case report. Pediatr Rheumatol. 2023;21(1):71. 10.1186/s12969-023-00859-y PMC10357603 37475052

[5] Durandy A, Kracker S. Increased activation of PI3 kinase-δ predisposes to B-cell lymphoma. Blood. 2020;135(9):638–43. 31942637 10.1182/blood.2019002072

[6] Coulter TI, Chandra A, Bacon CM, Babar J, Curtis J, Screaton N, et al. Clinical spectrum and features of activated phosphoinositide 3-kinase δ syndrome: A large patient cohort study. J Allergy Clin Immunol. 2017;139(2):597–e6064. 27555459 10.1016/j.jaci.2016.06.021 PMC5292996

[7] Pham MN, Cunningham-Rundles C. Evaluation of lymphoproliferative disease and increased risk of lymphoma in activated phosphoinositide 3 kinase delta syndrome: A case report with discussion. Front Pediatr. 2018;6:402. 10.3389/fped.2018.00402 PMC6305443 30619796

[8] Mauz-Körholz C, Hasenclever D, Dörffel W, Ruschke K, Pelz T, Voigt A, et al. Procarbazine-Free OEPA-COPDAC chemotherapy in boys and standard OPPA-COPP in girls have comparable effectiveness in pediatric hodgkin’s lymphoma: the GPOH-HD-2002 study. J Clin Oncol. 2010;28(23):3680–6. 20625128 10.1200/JCO.2009.26.9381

[9] Wang W, Min Q, Lai N, Csomos K, Wang Y, Liu L et al. Cellular mechanisms underlying B cell abnormalities in patients with Gain-of-Function mutations in the PIK3CD gene. Front Immunol. 2022;13:890073. 10.3389/fimmu.2022.890073 PMC9253290 35799777

[10] Thauland TJ, Pellerin L, Ohgami RS, Bacchetta R, Butte MJ. Case study: mechanism for increased follicular helper T cell development in activated PI3K delta syndrome. Front Immunol. 2019;10:753. 10.3389/fimmu.2019.00753 PMC6473200 31031754

[11] Takeda AJ, Zhang Y, Dornan GL, Siempelkamp BD, Jenkins ML, Matthews HF, et al. Novel PIK3CD mutations affecting N-terminal residues of p110δ cause activated PI3Kδ syndrome (APDS) in humans. J Allergy Clin Immunol. 2017;140(4):1152–e115610. 28414062 10.1016/j.jaci.2017.03.026 PMC5632585

[12] Lucas CL, Kuehn HS, Zhao F, Niemela JE, Deenick EK, Palendira U, et al. Dominant-activating germline mutations in the gene encoding the PI(3)K catalytic subunit p110δ result in T cell senescence and human immunodeficiency. Nat Immunol. 2014;15(1):88–97. 24165795 10.1038/ni.2771 PMC4209962

[13] Rivalta B, Amodio D, Milito C, Chiriaco M, Di Cesare S, Giancotta C et al. Case report: EBV chronic infection and lymphoproliferation in four APDS patients: the challenge of proper Characterization, therapy, and Follow-Up. Front Pediatr. 2021;9:703853. 10.3389/fped.2021.703853 PMC8448282 34540765

[14] Michalovich D, Nejentsev S. Activated PI3 kinase delta syndrome: from genetics to therapy. Front Immunol. 2018;9703853. 10.3389/fimmu.2018.00369 PMC5835040 29535736

[15] Stewart O, Gruber C, Randolph HE, Patel R, Ramba M, Calzoni E, et al. Monoallelic expression can govern penetrance of inborn errors of immunity. Nature. 2025;637(8048):1186–97. 39743591 10.1038/s41586-024-08346-4 PMC11804961

[16] Wang Y, Wang W, Liu L, Hou J, Ying W, Hui X, et al. Report of a Chinese cohort with activated phosphoinositide 3-Kinase δ syndrome. J Clin Immunol. 2018;38(8):854–63. 30499059 10.1007/s10875-018-0568-x

[17] Richards S, Aziz N, Bale S, Bick D, Das S, Gastier-Foster J, et al. Standards and guidelines for the interpretation of sequence variants: a joint consensus recommendation of the American college of medical genetics and genomics and the association for molecular pathology. Genet Sci. 2015;17(5):405–24. 10.1038/gim.2015.30 PMC4544753 25741868

[18] Stone EL, Pepper M, Katayama CD, Kerdiles YM, Lai CY, Emslie E, et al. ICOS coreceptor signaling inactivates the transcription factor FOXO1 to promote Tfh cell differentiation. Immunity. 2015;42(2):239–51. 25692700 10.1016/j.immuni.2015.01.017 PMC4334393

[19] Ma Y, Bao Y, Zheng M. Epstein–Barr virus-associated B-cell lymphoproliferative disorder meeting the definition of CAEBV B cell disease: a case report. BMC Infect Dis. 2023;23(1):453. 37420238 10.1186/s12879-023-08430-6 PMC10329294

[20] Roughan JE, Thorley-Lawson DA. The intersection of Epstein-Barr virus with the germinal center. J Virol. 2009;83(8):3968–76. 19193789 10.1128/JVI.02609-08 PMC2663245

