# Supplementary material for: APDS in a 3-year-old boy presenting with EBV viremia and hodgkin lymphoma associated with a novel germline heterozygous variant in PIK3CD and with characteristic immune phenotype but no upregulation of the T cell mTOR pathway
Source: Allergy Asthma Clin Immunol. 2026 Mar 17;22:23. doi: 10.1186/s13223-026-01014-4 (PMC13107807; doi:10.1186/s13223-026-01014-4)
Supplement: Supplementary file 1 — Supplementary Material 1 [file 13223_2026_1014_MOESM1_ESM.pdf]

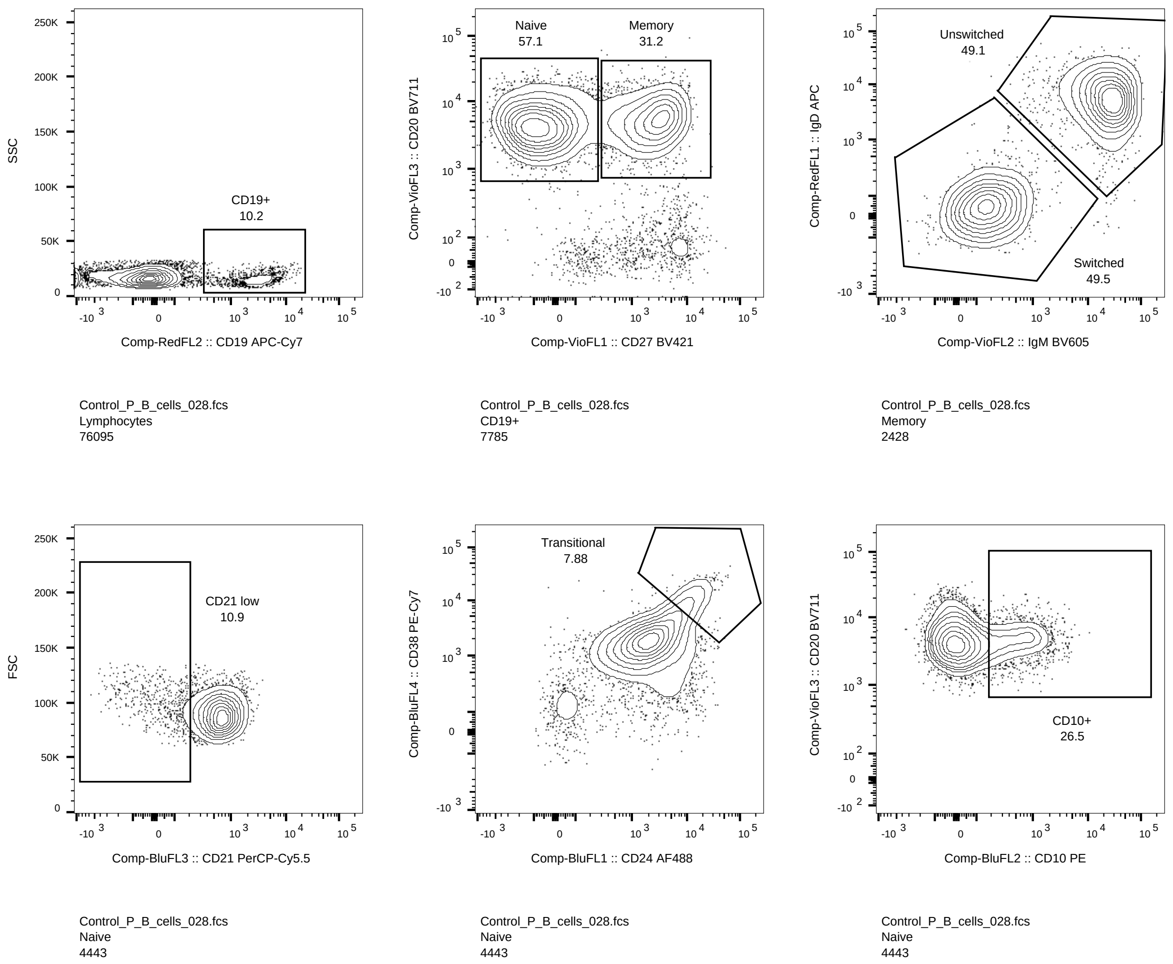
SSC
CD19+
10.2
Comp-RedFL2 :: CD19 APC-Cy7
Control_P_B_cells_028.fcs
Lymphocytes
76095
Comp-VioFL3 :: CD20 BV711
Naive
57.1
Memory
31.2
Comp-VioFL1 :: CD27 BV421
Control_P_B_cells_028.fcs
CD19+
7785
Comp-RedFL1 :: IgD APC
Unswitched
49.1
Switched
49.5
Comp-VioFL2 :: IgM BV605
Control_P_B_cells_028.fcs
Memory
2428
FSC
CD21 low
10.9
Comp-BluFL3 :: CD21 PerCP-Cy5.5
Control_P_B_cells_028.fcs
Naive
4443
Comp-BluFL4 :: CD38 PE-Cy7
Transitional
7.88
Comp-BluFL1 :: CD24 AF488
Control_P_B_cells_028.fcs
Naive
4443
Comp-VioFL3 :: CD20 BV711
CD10+
26.5
Comp-BluFL2 :: CD10 PE
Control_P_B_cells_028.fcs
Naive
4443

Control

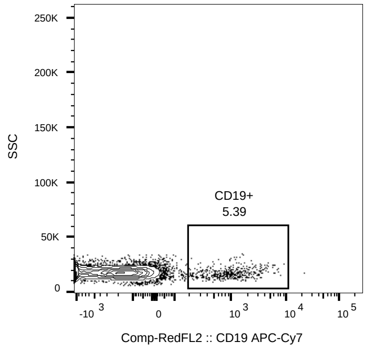

UCLA_416_B_cells_003.fcs
Lymphocytes
81188

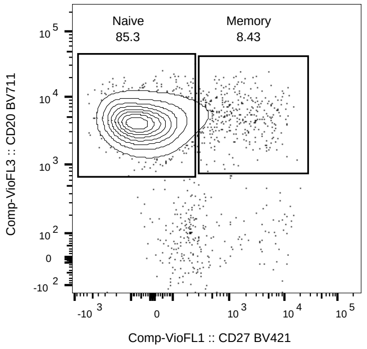

UCLA_416_B_cells_003.fcs
CD19+
4378

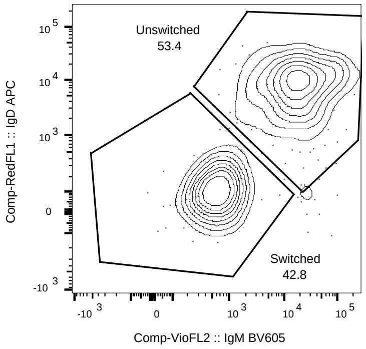

UCLA_416_B_cells_003.fcs
Memory
369

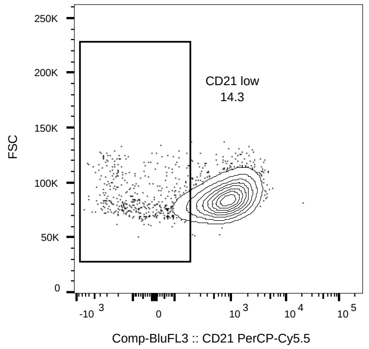

UCLA_416_B_cells_003.fcs
Naive
3733

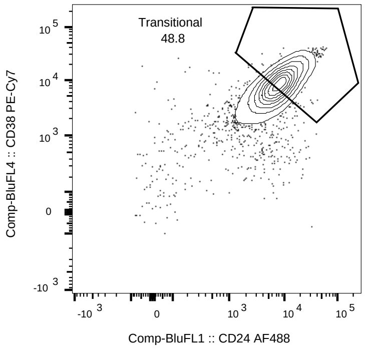

UCLA_416_B_cells_003.fcs
Naive
3733

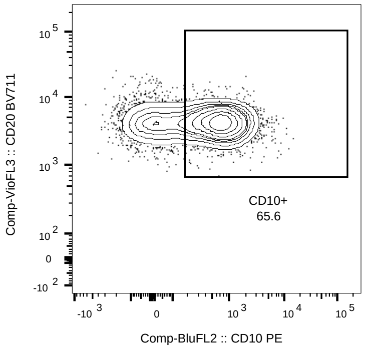

UCLA_416_B_cells_003.fcs
Naive
3733

UCLA 416

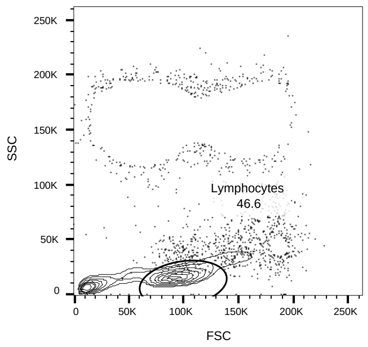

Control_P_T_cells_006.fcs
Ungated
97910

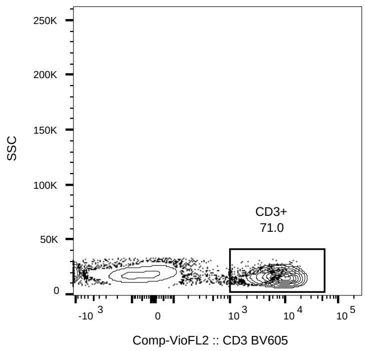

Control_P_T_cells_006.fcs
Lymphocytes
45595

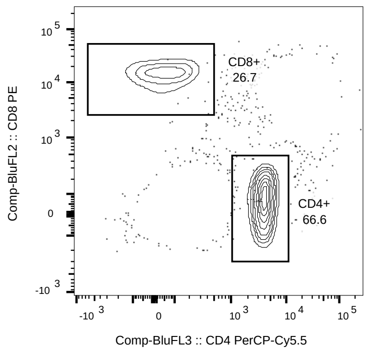

Control_P_T_cells_006.fcs
CD3+
32370

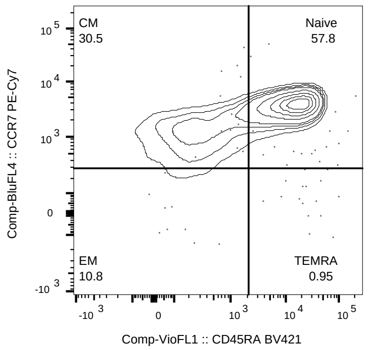

Control_P_T_cells_006.fcs
CD4+
21547

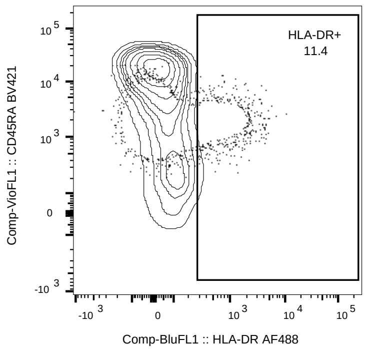

Control_P_T_cells_006.fcs
CD4+
21547

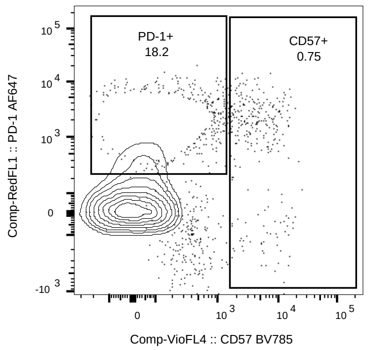

Control_P_T_cells_006.fcs
CD4+
21547

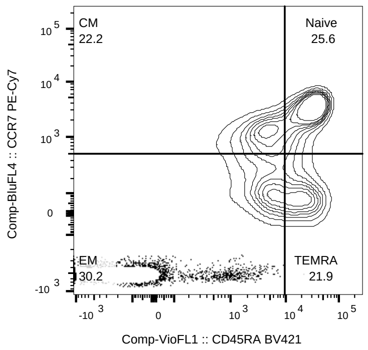

Control_P_T_cells_006.fcs
CD8+
8640

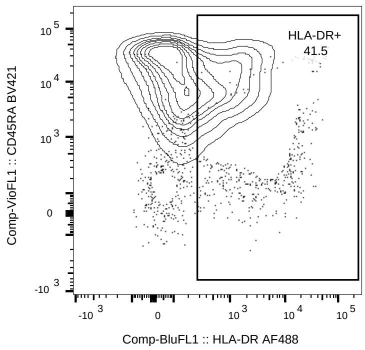

Control_P_T_cells_006.fcs
CD8+
8640

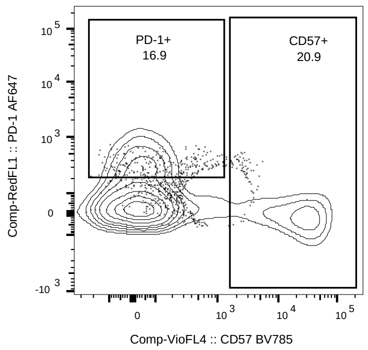

Control_P_T_cells_006.fcs
CD8+
8640

Control

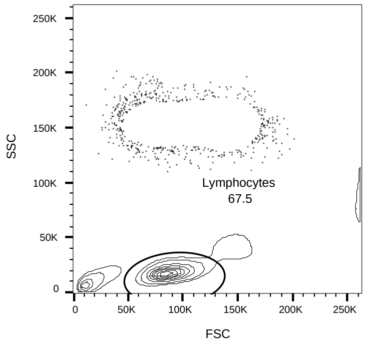

UCLA_416_T_cells_001.fcs
Ungated
90850

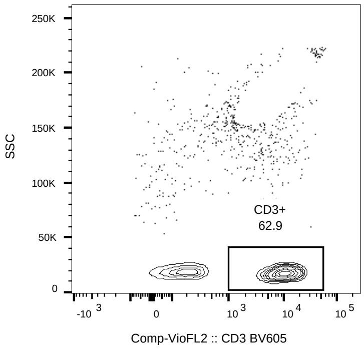

UCLA_416_T_cells_001.fcs
Lymphocytes
61359

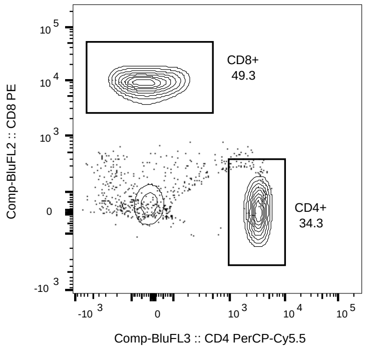

UCLA_416_T_cells_001.fcs
CD3+
38609

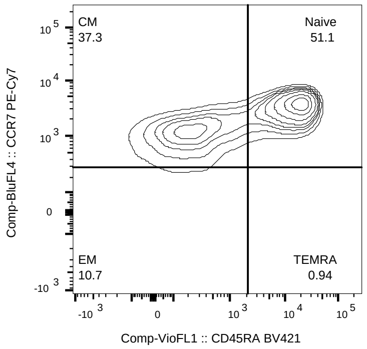

UCLA_416_T_cells_001.fcs
CD4+
13239

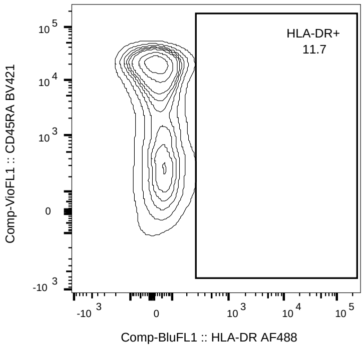

UCLA_416_T_cells_001.fcs
CD4+
13239

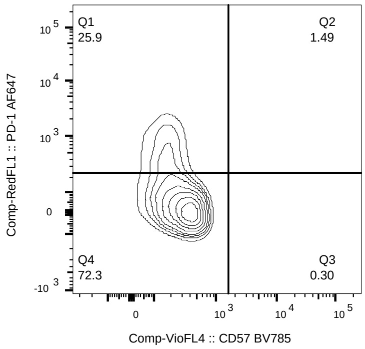

UCLA_416_T_cells_001.fcs
CD4+
13239

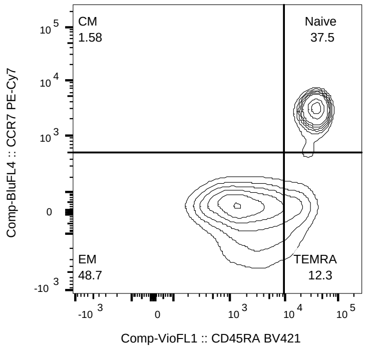

UCLA_416_T_cells_001.fcs
CD8+
19022

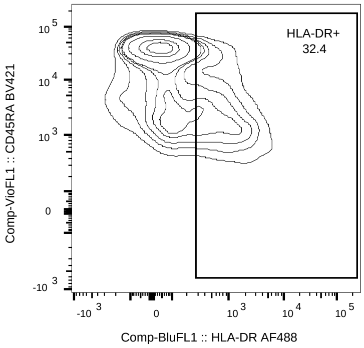

UCLA_416_T_cells_001.fcs
CD8+
19022

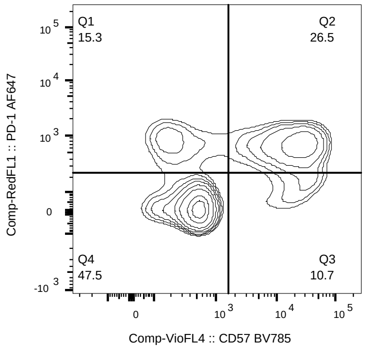

UCLA_416_T_cells_001.fcs
CD8+
19022

UCLA 416

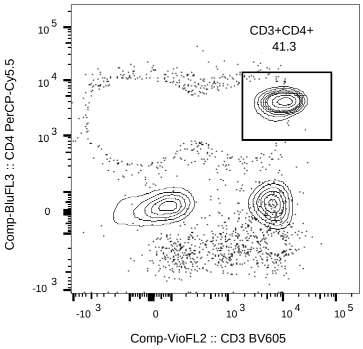

Control_P_Th_panel_017.fcs
Lymphocytes
56046

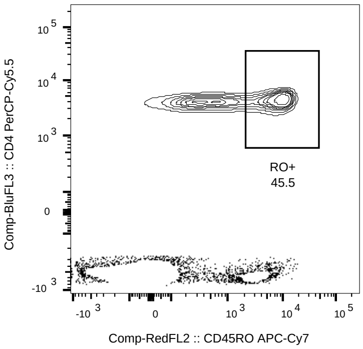

Control_P_Th_panel_017.fcs
CD3+CD4+
23153

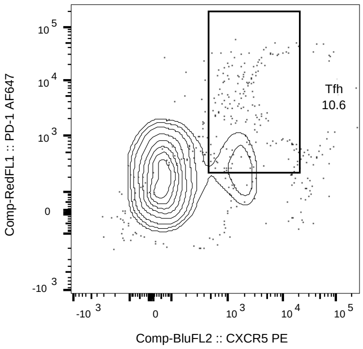

Control_P_Th_panel_017.fcs
RO+
10531

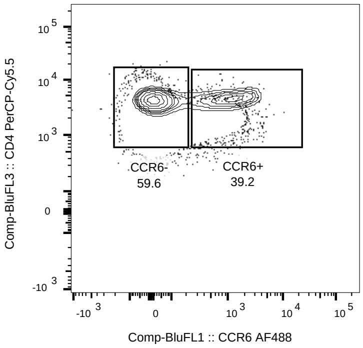

Control_P_Th_panel_017.fcs
RO+
10531

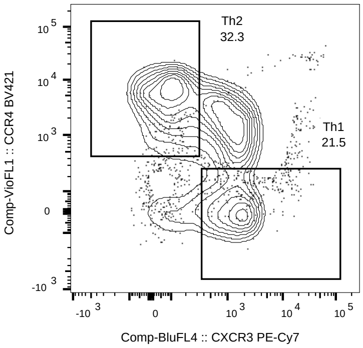

Control_P_Th_panel_017.fcs
CCR6-
6275

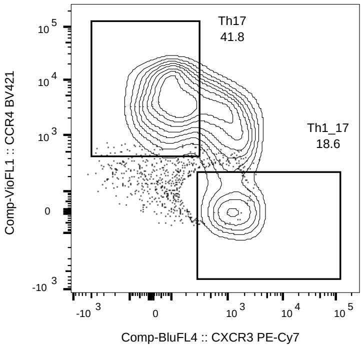

Control_P_Th_panel_017.fcs
CCR6+
4127

Control

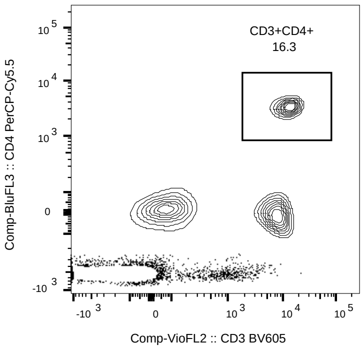

UCLA_416_Th_panel_002.fcs
Lymphocytes
155402

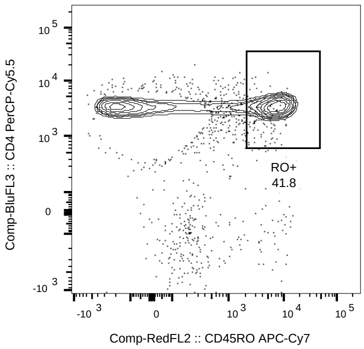

UCLA_416_Th_panel_002.fcs
CD3+CD4+
25263

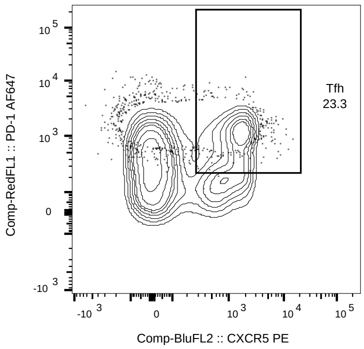

UCLA_416_Th_panel_002.fcs
RO+
10559

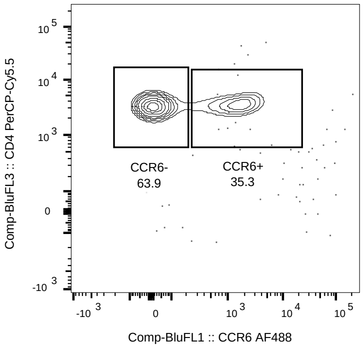

UCLA_416_Th_panel_002.fcs
RO+
10559

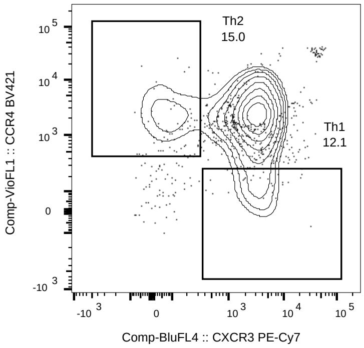

UCLA_416_Th_panel_002.fcs
CCR6-
6751

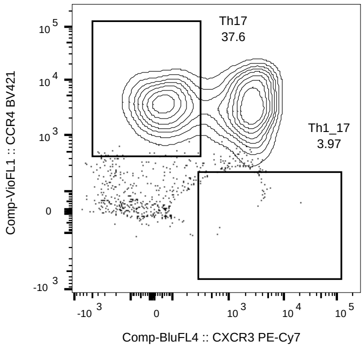

UCLA_416_Th_panel_002.fcs
CCR6+
3726

UCLA 416

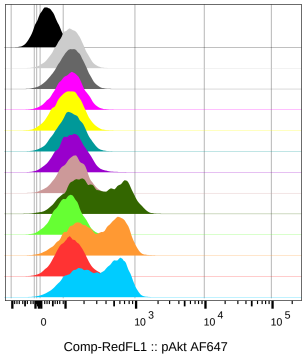

	Sample Name	Subset Name	Geometric Mean : Comp-RedFL1
	Isotypes_001.fcs	CD4+	34.7
	Control_Q_unstim_002.fcs	CD4+	122
	Control_Q_stim_003.fcs	CD4+	128
	Control_P_unstim_004.fcs	CD4+	124
	Control_P_stim_005.fcs	CD4+	118
	UCLA_416_unstim_006.fcs	CD4+	128
	UCLA_416_stim_007.fcs	CD4+	133
	PI3K_110_01_unstim_008.fcs	CD4+	137
	PI3K_110_01_stim_009.fcs	CD4+	297
	PI3K_110_01_unstim_leni_010.fcs	CD4+	114
	PI3K_110_01_stim_leni_011.fcs	CD4+	293
	PI3K_100_01_unstim_leni_012.fcs	CD4+	129
	PI3K_100_01_stim_leni_013.fcs	CD4+	304

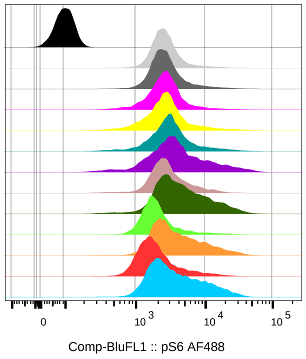

	Sample Name	Subset Name	Geometric Mean : Comp-BluFL1
	Isotypes_001.fcs	CD4+	100
	Control_Q_unstim_002.fcs	CD4+	2648
	Control_Q_stim_003.fcs	CD4+	2736
	Control_P_unstim_004.fcs	CD4+	2564
	Control_P_stim_005.fcs	CD4+	2676
	UCLA_416_unstim_006.fcs	CD4+	3079
	UCLA_416_stim_007.fcs	CD4+	3802
	PI3K_110_01_unstim_008.fcs	CD4+	2874
	PI3K_110_01_stim_009.fcs	CD4+	4408
	PI3K_110_01_unstim_leni_010.fcs	CD4+	2091
	PI3K_110_01_stim_leni_011.fcs	CD4+	3854
	PI3K_100_01_unstim_leni_012.fcs	CD4+	2004
	PI3K_100_01_stim_leni_013.fcs	CD4+	3692

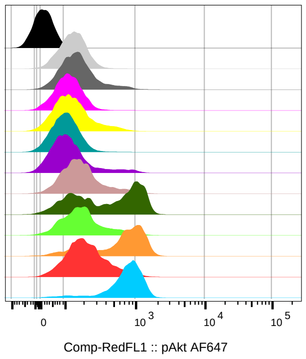

	Sample Name	Subset Name	Geometric Mean : Comp-RedFL1
	Isotypes_001.fcs	CD19+	21.0
	Control_Q_unstim_002.fcs	CD19+	141
	Control_Q_stim_003.fcs	CD19+	170
	Control_P_unstim_004.fcs	CD19+	118
	Control_P_stim_005.fcs	CD19+	140
	UCLA_416_unstim_006.fcs	CD19+	105
	UCLA_416_stim_007.fcs	CD19+	129
	PI3K_110_01_unstim_008.fcs	CD19+	188
	PI3K_110_01_stim_009.fcs	CD19+	384
	PI3K_110_01_unstim_leni_010.fcs	CD19+	202
	PI3K_110_01_stim_leni_011.fcs	CD19+	569
	PI3K_100_01_unstim_leni_012.fcs	CD19+	225
	PI3K_100_01_stim_leni_013.fcs	CD19+	693

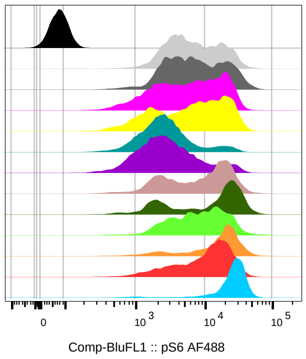

	Sample Name	Subset Name	Geometric Mean : Comp-BluFL1
	Isotypes_001.fcs	CD19+	78.1
	Control_Q_unstim_002.fcs	CD19+	6468
	Control_Q_stim_003.fcs	CD19+	6932
	Control_P_unstim_004.fcs	CD19+	5405
	Control_P_stim_005.fcs	CD19+	5749
	UCLA_416_unstim_006.fcs	CD19+	2642
	UCLA_416_stim_007.fcs	CD19+	2839
	PI3K_110_01_unstim_008.fcs	CD19+	7400
	PI3K_110_01_stim_009.fcs	CD19+	9669
	PI3K_110_01_unstim_leni_010.fcs	CD19+	7905
	PI3K_110_01_stim_leni_011.fcs	CD19+	12626
	PI3K_100_01_unstim_leni_012.fcs	CD19+	9091
	PI3K_100_01_stim_leni_013.fcs	CD19+	21706

UCLA 416 - p110δ - Val20Ile

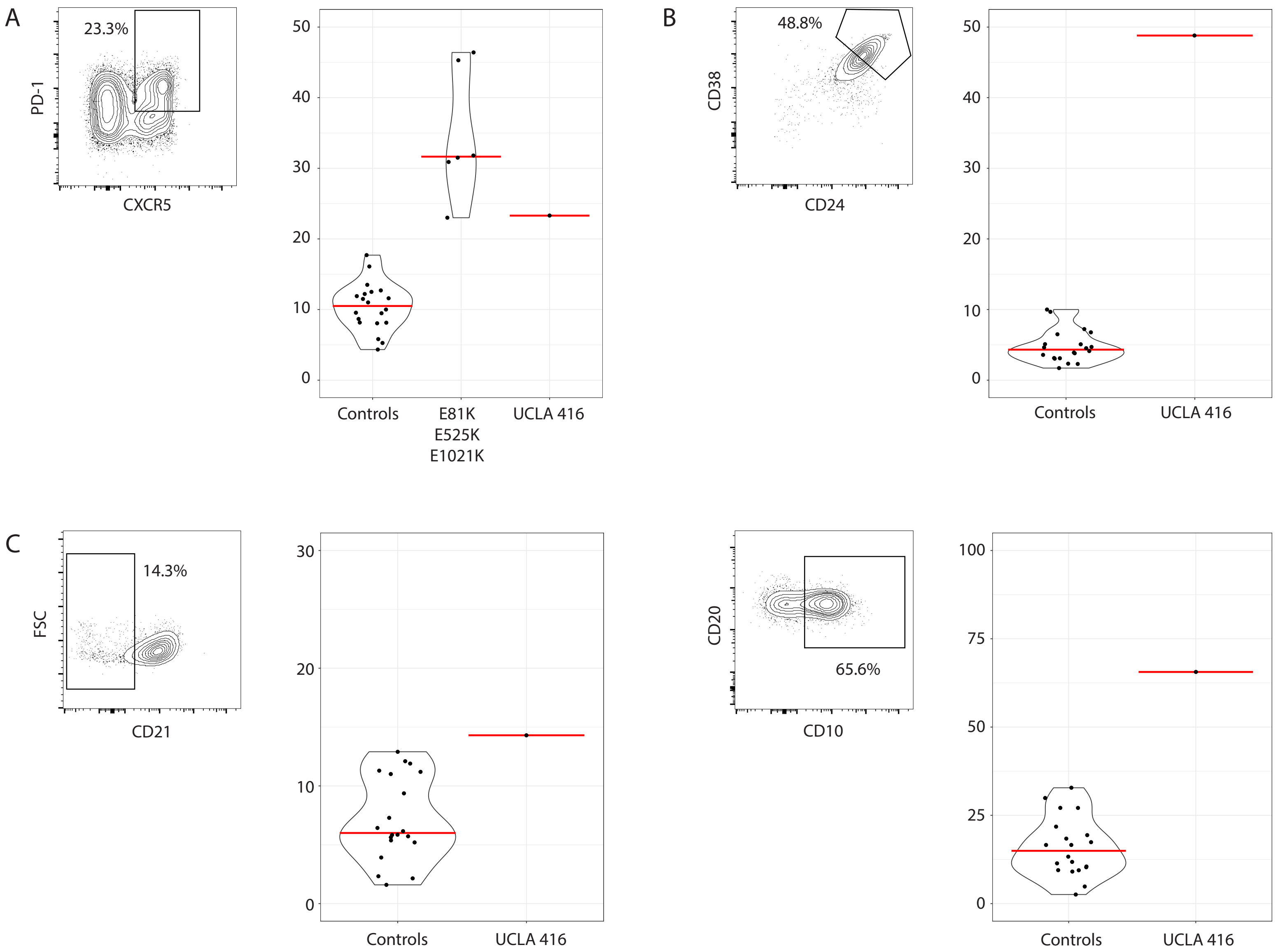

(A) Percentage of T follicular helper (Tfh) cells among CD3$^+$CD4$^+$CD45RO$^+$ cells was determined by flow cytometry of PBMCs. The result is compared to healthy controls and known APDS patients. **(B-D)** Percentage of CD24^{++}CD38^{++} transitional (B), CD21 low immature (C), or CD10$^+$ cells among CD19$^+$CD20$^+$CD27$^-$ B cells. Results are compared to healthy controls.
